# In the September 2016 issue

**DOI:** 10.1590/S1980-5764-2016DN1003001

**Published:** 2016

**Authors:** Ricardo Nitrini

In this issue we are publishing one review, eight original papers, four case reports and one history note. 

Studart-Neto and Nitrini reviewed the state-of-the-art concerning subjective cognitive decline in the elderly. The dark boundaries between the impact of normal aging and early signs of Alzheimer's disease (AD) may be illuminated through novel methods, including biomarkers, and neuroimaging advances. 

Fukushima et al. systematically reviewed studies on the effects of cognitive stimulation on the neuropsychiatric symptoms in AD, mainly apathy, depression and anxiety. The authors concluded that cognitive stimulation programs are an effective therapeutic alternative for these otherwise difficult-to-manage symptoms of AD. 

Holanda and Almondes performed a systematic review to evaluate the role of age-related sleep changes in executive functions (EF). Several markers of poor sleep quality were associated with worse EF scores.

Mota et al. investigated the association of low education with delirium in adults at an Emergency Department over a 6-month period. Delirium was more frequent in the aged and in low-educated individuals. 

Schelp et al. evaluated the relationships between episodic memory, age, and education in patients with Parkinson Disease. Patients with episodic memory impairment were older and had low performance on other screening tests, supporting reports of a wide spectrum of neuropsychological performance impairment in PD. 

Ortiz et al. investigated the presence of speech disorders in PD, particularly sensorimotor disorders of programming, in addition to execution disorders. As a group, patients with PD had worse performance on all sensorimotor speech tasks.

Roman et al. translated and adapted the Memory Binding Test (MBT) to estimate its diagnostic accuracy as a tool for early detection of amnestic mild cognitive impairment (aMCI) in Argentina. They concluded that the Argentinian version of the MBT is a useful tool for the detection of aMCI.

Beber et al. compared performances on two clock drawing tests (CDT) of patients with MCI or dementia. One CDT was freely-drawn whereas the other was an incomplete-copy where only the hands had to be drawn. The free-drawn CDT version was more sensitive for detecting mild/early cognitive impairment.

Mantovani-Nagaoka et al. evaluated the performance of healthy subjects on a limb apraxia battery and analysed the influence of gender, age, and education on it. They found an important influence of education level on the outcome of limb apraxia tests. 

Passos et al. reported a mother and her only daughter with incapacitating behavioral manifestations of frontal lobe dysfunction associated with epilepsy, and discussed the likelihood of a hitherto unreported genetic condition. 

Brucki et al. reported the case of a 63-year-old woman with an arteriovenous malformation (AVM) associated with progressive dementia. The plausible causal relationship between AVM and dementia is discussed. 

Martinelli et al. reported a case of a 79-year-old woman with musical hallucination (hearing a sung National anthem) without hearing loss, cognitive impairment or known psychiatric condition. Neuroleptics led to complete remission.

Costa et al. reported a case of pantothenate kinase-associated neurodegeneration (PKAN) that presented with typical symptoms of Tourette syndrome. The severity and poor response to treatment led to further investigation and to the diagnosis of PKAN as a secondary cause of Tourettism.

Engelhardt in this history note outlined the discoveries of François Magendie and Hubert von Luschka, who described the median and lateral openings of the 4^th^ ventricle, subsequently named after them. The author also revealed that even after these discoveries, the existence of these holes was questioned by many anatomists. 

